# Learning curve in open groin hernia surgery: nationwide register-based study

**DOI:** 10.1093/bjsopen/zrad108

**Published:** 2023-10-26

**Authors:** Olof Bladin, Nathalie Young, Jonas Nordquist, Joy Roy, Hans Järnbert-Pettersson, Gabriel Sandblom, Jenny Löfgren

**Affiliations:** Department of Molecular Medicine and Surgery, Karolinska Institute, Stockholm, Sweden; Acute and Trauma Surgery, Karolinska University Hospital, Stockholm, Sweden; Department of Clinical Science and Education, Södersjukhuset Karolinska Institute, Stockholm, Sweden; Department of Medicine (Huddinge), Karolinska Institute, Stockholm, Sweden; Department of Molecular Medicine and Surgery, Karolinska Institute, Stockholm, Sweden; Department of Clinical Science and Education, Södersjukhuset Karolinska Institute, Stockholm, Sweden; Department of Clinical Science and Education, Södersjukhuset Karolinska Institute, Stockholm, Sweden; Department of Surgery, Södersjukhuset, Stockholm, Sweden; Department of Molecular Medicine and Surgery, Karolinska Institute, Stockholm, Sweden

## Abstract

**Background:**

Current recommendations regarding the number of open groin hernia repairs that surgical trainees are required to perform during their residency are arbitrarily defined and vary between different curricula. This register-based study sought to investigate the learning curve of surgeons performing open anterior mesh repair for groin hernia by assessing hernia recurrence rates, surgical complications and operating times in relation to the number of procedures performed.

**Method:**

Nationwide data on open anterior mesh repair for groin hernia performed by surgical residents were collected from the Swedish Hernia Register between 2005 and 2020. The data were analysed in a cohort undergoing procedures carried out by surgeons performing their first registered repair as resident general surgeons. Repairs by surgeons with fewer than 30 repairs were excluded.

**Results:**

A total of 38 845 repairs carried out by 663 surgeons were included. Operation time decreased with increasing number of performed procedures, mean (s.d.) operation time was 79 (26) min for the first 15 procedures and 60 (23) min after 241 procedures (*P* <0.001). A turning point where complication rates began to decrease was seen after 60 procedures. Complication rates were 3.6 per cent (396 of 10 978) for procedures 31–60 and 2.7 per cent (157 of 5 798) for procedures 61–120 (*P* = 0.002). There was no significant relationship between the number of procedures performed and the rate of operation on for recurrence (*P* = 0.894).

**Conclusion:**

Sixty performed procedures during surgical residency is a reasonable target for achieving competency to perform open anterior mesh repair for groin hernia safely without supervision.

## Teaser

Minimum operation numbers for open anterior inguinal hernia mesh repair are set arbitrarily and vary between different curricula. Hernia recurrence rates, surgical complications and operation times were compared with the operation volumes of surgical trainees in the Swedish Hernia Register to investigate the learning curve for the procedure.

## Introduction

The science of skill acquisition has been troubling scholars for decades. To better understand the process of learning, Dreyfus and Dreyfus developed a model in which a learner progresses through different steps of learning, from novice to expert level^1^. Although individual variations exist, frequent repetitions of a given task clearly play an essential role in progressing to high-performance levels^2,3^. In surgery, knowledge of the learning curve of a procedure is essential for designing training programmes, as well as to understand how much training is needed before a trainee can be expected to perform a procedure independently.

Groin hernia is a very common surgical condition globally, and an estimated 20 million groin hernia repairs are performed around the world annually^4^. Each year, approximately 16 000 groin hernia repairs are carried out in Sweden^5^, and in the USA, the number of annual groin hernia repairs exceeds 800 000^6^. General surgeons are expected to be able to perform open anterior mesh repair for groin hernia independently after residency^7–9^.

Several frameworks have been developed to assess residents’ competency in surgical procedures^10–12^. Residents are likely to reach the goals of these frameworks after varying intervals of training. Notwithstanding individual variations, national surgical training curricula often provide minimum operation numbers, however, national requirements vary^13^. According to Swedish guidelines, a resident is expected to complete at least 30 hernia operations^8^. In comparison, the Joint Committee on Surgical Training in the UK requires at least 60 groin hernia operations upon completing residency^9^. These differences indicate that there is no reliable basis for the different recommendations. The international guidelines on groin hernia management provided by the European Hernia Society recommend no specific minimum operation numbers but rather recommend that each trainee should be supervised until they reach proficiency levels^14^. As recommendations vary, it is important to identify the surgical volume after which a trainee can be expected to operate independently. A recent report showed that American surgery residents had completed, on average, 87 groin hernia repairs upon finishing their residency. In the UK, the average was 117 procedures^15,16^.

The authors' hypothesis was that with an increasing number of procedures, there could be a certain threshold, where operation time, surgical complications and potentially operation on for recurrent hernia would decrease, indicating the existence of a learning curve for open anterior mesh repair of groin hernias.

Therefore, this register-based study aimed to investigate whether there was an association between the number of operations and operation time, surgical complications and the risk of operation for recurrence in open anterior mesh repair for groin hernia.

## Method

### Study design

This observational study analysed routinely collected health data from the Swedish Hernia Register to examine the relationship between surgical complications, operation time and the accumulated number of procedures performed by surgeons who performed their first registered open anterior mesh repair when they were resident surgeons.

### Study population

The study population included all resident surgeons who performed their first registered open anterior mesh repair for groin hernia (that is any hernia in the groin area) between 2005 and 2020 in Sweden. The cohort included both elective and emergency procedures as well as both primary and recurrent hernias. To ensure that the learning curve was followed from the first repair carried out, the years 1992–2004 were treated as a wash-out interval during which repairs by surgeons performing their first repair were excluded. All repairs performed by surgeons already registered as specialists when carrying out the first repair, and repairs by surgeons performing fewer than 30 repairs, were also excluded. Repairs with extreme operation times (<10 min or >600 min) were assumed to be incorrectly registered and thus excluded.

### Data collection

Data were assembled from the Swedish Hernia Register, which has had national coverage since 1997^17^. More than 95 per cent of groin hernia repairs on adults in Sweden are registered in the Swedish Hernia Register^5^. In the register, each surgeon has a unique code at each healthcare unit where she or he works. If a surgeon performs hernia repairs in more than one healthcare unit, she or he will therefore have more than one code. Residents will not receive a new code if they partake in rotations at different hospitals during their residency training. The register does not routinely incorporate follow-up information, and complications will only be registered if they are discovered during the hospital stay or if patients seek healthcare. Recurrence of hernia is registered only if it is operated on. All follow-up data registered between 2005 and 2020 were included.

### Factors

The primary outcome of the study was surgical complications (bleeding, infection, visceral injury or acute reoperation on). A standardized definition for complications was not established, allowing individual surgeons to determine their own criteria for identifying complications. Secondary outcomes were operation time and operation on for recurrence. In general, operation time is defined as the duration from the incision to the final stitch; nevertheless, it is plausible that certain operation times were not reported in accordance with this specific definition. The independent factors used for the classification trees were the ASA physical status classification, supervised procedure, hernia anatomy, patient age and type of procedure (elective or acute). Supervised procedure was defined as one or more surgeons being registered as a co-surgeon. Hernia anatomy was defined as medial, lateral or other, which refers to combined hernias, pantaloon hernias, sportsman's hernias, femoral hernias and other uncategorizable descriptions of hernias. The independent factors were patient related or surgeon related; the purpose of including patient-related factors was to reduce confounding factors when attempting to determine how surgeon-related factors influence the outcomes.

### Statistical methods

Kaplan–Meier curves and the corresponding Log Rank test were used to assess the cumulative incidence of operation on for recurrent hernia and study differences across varying levels of procedure count. A two-sample *t* test was used to compare operation times and a chi-square test was used to compare surgical complications. Classification trees were used to identify factors associated with each of the outcomes: surgical complications and operation time. These analyses were also used to stratify groups of patients according to risk of complication as well as operation time. The advantages with classification trees are that they detect interactions in data and are easy to interpret because data splits up in distinct groups. The groups represent individiuals which are similar within the nodes and different between the nodes. The chi-square Automatic Interaction Detection (CHAID) algorithm was used to build the trees^18^. A CHAID analysis studies the cross-tabulations between each of the independent factors and the outcome and tests for significance using a chi-square test. Factors with more than two categories are compared, and categories that are similar with respect to the outcome are merged together. Missing data are treated as a single category for each factor, which implies that missing data can be compared with categories with valid data with respect to the outcome. The independent factors were categorized as presented in *Table 1*. The resulting groups were split until one of the following stop criteria was reached: a maximum depth for the tree equal to three, a minimum group size for child nodes of 100 patients or a Bonferroni adjustment split of less than 0.05 to continue to grow the tree. All analyses were performed using SPSS version 28 and R studio 2022.02.2.

**Table 1:** Descriptive statistics of open anterior mesh repairs, surgical complications and operation time in minutes

	Count, *n* (%)	Complication, *n* (%)	Mean (s.d.) operation time
**Sex**			
Male	37 625 (96. 9)	1181 (3.1)	74 (27)
Female	1220 (3.1)	23 (1.9)	66 (25)
**ASA** *			
ASA I	17 160 (44.2)	338 (2.0)	72 (26)
ASA II	16 589 (42.7)	534 (3.2)	75 (27)
ASA III	4919 (12.7)	322 (6.5)	77 (28)
ASA IV	176 (0.5)	9 (5.1)	81 (33)
ASA V	1 (0.0)	1 (100)	74
**Supervision**†			
No supervision	19 050 (49.0)	666 (3.5)	70 (26)
Supervision	19 795 (51.0)	538 (2.7)	78 (27)
**Patient age (years)**			
≤ 60	13 825 (35.6)	343 (2.5)	73 (26)
60–75	16 802 (43.3)	465 (2.8)	75 (27)
>75	8218 (21.2)	396 (4.8)	75 (27)
**Number of procedures**‡			
1–15	9884 (25.4)	298 (3.0)	79 (26)
16–30	9603 (24.7)	324 (3.4)	78 (27)
31–60	10 978 (12.7)	396 (3.6)	74 (27)
61–120	5798 (14.9)	157 (2.7)	66 (26)
121–240	1637 (4.2)	25 (1.5)	64 (24)
241–	945 (2.4)	4 (0.4)	60 (23)
**Type of surgery**			
Acute	1434 (3.7)	89 (6.2)	83 (33)
Elective	37 411 (96.3)	1115 (3.0)	74 (27)
**Hernia anatomy**			
Lateral	21 490 (55.3)	712 (3.3)	75 (27)
Medial	13 158 (33.9)	340 (2.6)	70 (26)
Other§	4127 (10.6)	147 (3.6)	82 (29)
Missing	70 (0.2)		

^*^ASA physical status classification. †Registered co-surgeon in the Swedish Hernia Register. ‡Number of open anterior mesh repairs previously performed by the surgeon at the time of surgery. §Combined hernias, pantaloon hernias, sportsman’s hernias, femoral hernias and other uncategorizable descriptions of hernias.

### Ethics

The study was approved by the Swedish Ethical Review Authority, Dnr 2021–03331.

## Results

**Fig. 1:**
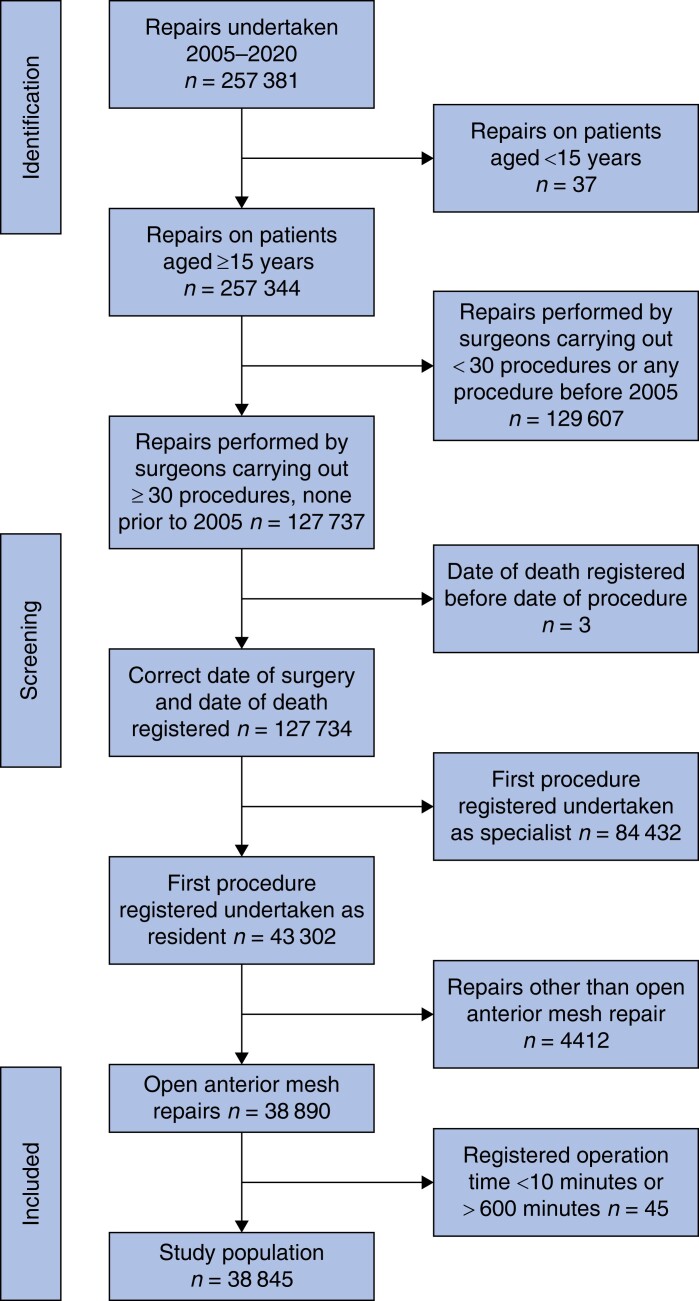
Study flow chart

Between 1 January, 2005 and 31 December, 2020, 257 381 groin hernia repairs were performed. The process of obtaining the study cohort for the present study is shown in *Fig. 1*. Altogether, 38 845 repairs were carried out by 663 surgeons registered as residents when performing their first open anterior mesh repair. Of these, 51 per cent (19 795 of 38 845) were performed under supervision and 49.0 per cent (19 050 of 38 845) without supervision (*Table 1*). A total of 96.9 per cent (37 625) of procedures were carried out in male patients and 3.1 per cent (1220) in females. The patients’ mean age was 63.1 years, and 86.9 per cent (33 749) had an ASA classification score of I or II.

**Fig. 2:**
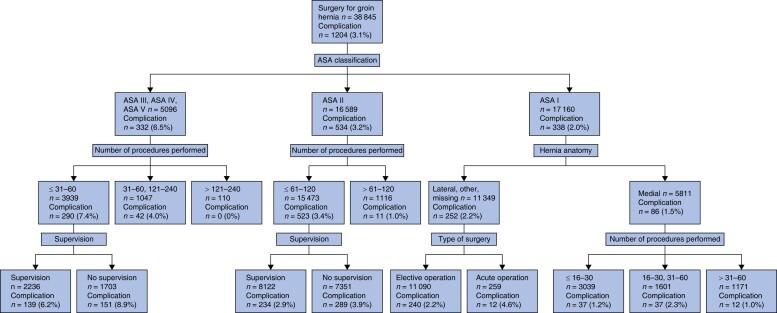
Tree analysis of surgical complications The figure shows that 3.1 per cent of all patients suffered from surgical complications. The highest complication rate (8.9 per cent) was found in patients with an ASA score of III to V where the procedures were performed by unsupervised residents with experience of 60 procedures or fewer.

Surgical complications were registered for 3.1 per cent (1204 of 38 845) of patients (*Table 1*). The factor with the strongest association to surgical complications was ASA score (*Fig. 2*). In the ASA I group, 2.0 per cent (338 of 17 498) of patients developed surgical complications, compared with 6.5 per cent (332 of 5096) in the ASA III—ASA IV group. For patients with an ASA score higher than one, factors associated with lower complication rates were a larger number of procedures performed and supervised surgery. For patients with ASA I, the presence of a medial hernia defect was associated with lower complication rates. For patients with ASA I and hernia anatomy other than medial, an elective procedure was associated with lower complication rates.

**Fig. 3:**
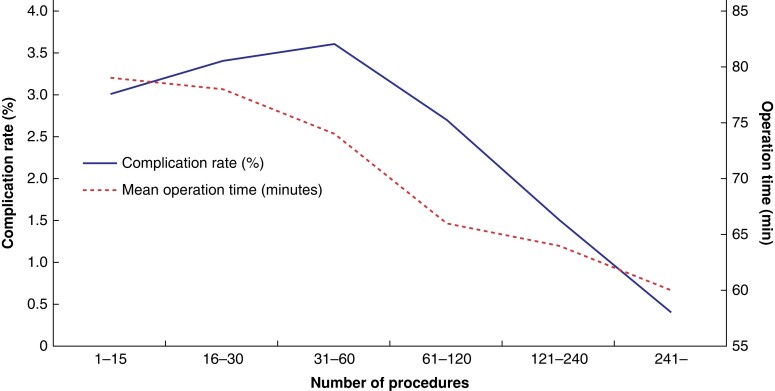
Number of procedures performed by the surgical residents in relation to surgical complications and mean operation time Complication rates are shown as percentages and mean operation time is shown in minutes (s.d.). Group 1–15: complication rate = 3.0 per cent (298 of 9884), mean operation time = 79 (26). Group 16–30: complication rate = 3.4 per cent (324 of 9603), mean operation time = 78 (27). Group 31–60: complication rate = 3.6 per cent (396 of 10 978), mean operation time = 74 (27). Group 61–120: complication rate = 2.7 per cent (157 of 5798), mean operation time = 66 (26). Group 121–240: complication rate = 1.5 per cent (25 of 1637), mean operation time = 64 (24). Group 241– : complication rate = 0.4 per cent (4 of 945), mean operation time = 60 (23).

**Fig. 4:**
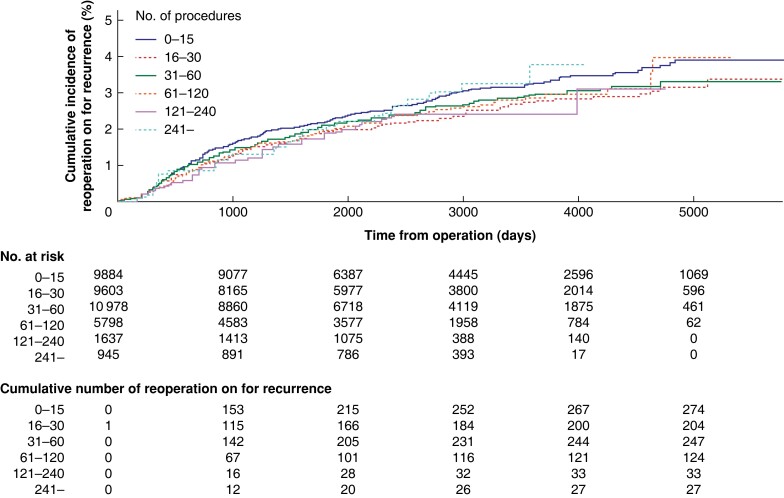
Cumulative incidence of reoperation on for recurrent hernia Log Rank test (*P* = 0.894) shows no evidence for differences between operators with a different number of earlier procedures.

Complications occurred in 2.7 per cent (538 of 19 795) of patients who had undergone an operation performed by a supervised surgical resident and in 3.5 per cent (666 of 19 050) of patients who had undergone an operation performed by an unsupervised resident (*Table 1*). The rate of surgical complications reached its highest point at 3.6 per cent (396 of 10 978) at 31–60 procedures performed, after which it decreased to 2.7 per cent (157 of 5798), for procedures 61–120 (*P* = 0.002) (*Fig. 3*). A total of 2.9 per cent (1132 of 38 845) of operations for recurrence was registered. The rate of operation on for recurrence was not associated with the number of procedures performed (*P* = 0.894) (*Fig. 4*).

The mean operation time was 74.1 min (s.d. 18.35). Operation time shortened with increasing number of procedures performed. Thus, the mean operation time for the first 15 procedures was 79 (s.d. 26) minutes, while after 241 procedures it decreased to 60 (s.d. 23) minutes (*P* <0.001) (*Fig. 3*). A tree analysis showed that the accumulated number of procedures performed was the factor with the strongest association with operation time (*Fig. S1*). At the next two levels of the tree analysis, supervision and hernia anatomy were the factors most strongly associated with operation time. The presence of a medial hernia defect and unsupervised procedures were associated with shorter operation times at different levels of the tree analysis (*Fig. S1*).

## Discussion

In this observational study based on the Swedish Hernia Register, the overall risk of surgical complications was 3.1 per cent. Surgical complications decreased after 60 procedures performed and complications were more frequent if a resident surgeon performed a procedure without supervision. However, a high ASA score was the factor with the strongest association with higher complication rates and neither number of procedures performed nor supervision were associated with complications for patients with ASA I and hernia anatomy other than medial, accounting for 29.2 per cent of all patients. For patients with ASA higher than one, complication rates were higher with a lower number of procedures performed and the absence of supervision. Thus, supervision and experience appear to matter more when operating on patients that already carry an increased risk of developing surgical complications. The mean operation time continuously decreased with increasing numbers of procedures performed.

A strength of this study is the high-quality data and the high cover rate of the Swedish Hernia Register, which made the present large study population possible^17^. This study has some limitations that should be noted. First, it should be highlighted that the Swedish Hernia Register only captures operations for hernia recurrence and not clinical recurrences, potentially leading to underreporting of the recurrence rate. Additionally, the inclusion of procedures carried out between 2005 and 2020 raises the possibility that patients who underwent surgery in the later years may still experience hernia recurrence requiring reoperation on. This is illustrated in *Fig. 4*, where a greater number of patients have been followed for an extended duration when operated on by surgeons with a lower procedure volume. Second, the Swedish Hernia Register does not capture quality of life or chronic pain, which are important outcomes after groin hernia surgery. Moreover, the use of ‘registered co-surgeon’ as a definition for a procedure being performed under supervision is a potential limitation, as the co-surgeon may not always represent a more experienced surgeon supervising the surgical trainee. It is, however, reasonable to assume that the majority of procedures involving more than one surgeon are carried out as supervised procedures with the purpose of education, as elective groin hernia surgery rarely requires several experienced surgeons. Another limitation of this study is that while each surgeon is assigned a unique code at each respective healthcare unit, the code no longer applies if the surgeon changes their place of employment. This means that if a surgeon changes employment and begins working at a different healthcare unit, their previous code will no longer be tracked in the Swedish Hernia Register. This limitation may obscure the outcome, as the data may not capture all the procedures performed by a given surgeon throughout their career. It is possible that some surgeons may have performed their first open anterior mesh repair outside of Sweden, and thus already gained experience as the main surgeon when her or his first procedure was registered in the Swedish Hernia Register. Furthermore, the proficiency of surgical residents in hernia surgery may be influenced by their experience in other surgical procedures. Nonetheless, the present study is constrained by the absence of consideration given to the experience gained from performing other procedures. However, this reflects the actuality of surgical residency programmes, where residents are exposed to learning multiple procedures concurrently. Although this study included a large sample size and utilized data from the Swedish Hernia Register, which has a high coverage rate, the findings may not be directly generalizable to healthcare systems and surgical training programmes that differ substantially from those in Sweden. Therefore, caution should be taken when applying these results to other settings, especially those where hernia repair is a primary focus of a surgical training programme.

The authors excluded procedures carried out by residents who had performed at least one procedure before 2005 and those by residents who had performed fewer than 30 procedures. This was done to minimize the risk of including surgeons who had already carried out hernia repairs before the Hernia Register started, at other units under other key numbers. Nevertheless, there may have been surgeons who had already acquired experience in hernia surgery, which may have obscured the results. Future studies that use routinely collected health data should consider strategies to mitigate this limitation, such as including data from multiple registers or developing methods to track surgeons across different healthcare units.

Patient safety should be the primary concern for any surgical intervention. This study shows that groin hernia repair performed by resident surgeons carries a low risk of surgical complications and that recurrence rates are comparable to those of specialist surgeons^19,20^. Some confounders, however, exist. The patients operated on were, in general, healthy men, with only a small group of patients in ASA class III and above. The procedures were mostly elective cases, which is associated with lower complication rates^21^. Additionally, the decrease in surgical complications was accompanied by a corresponding reduction in operation time, potentially exerting an influence on the incidence of complications^22^.

A recent study from the Danish Hernia Register found no significant difference in reoperation risk for recurrent groin hernia between supervised residents and specialists^23^. In a report from the Swedish Hernia Register, it was found that male patients are at greater risk of reoperation on for recurrent groin hernia when a surgical trainee exceeded a certain operation time, emphasizing the importance of structured supervision^24^. Another analysis showed that unsupervised junior residents had higher recurrence rates compared with senior residents and consultants^25^. Supervision varies between early and late training. The Ottawa Surgical Competency Operating Room Evaluation (O-SCORE) assesses surgical competency based on the level of supervision received during a procedure^11^. Novice residents require more direct supervision, while experienced residents may only need verbal directions. This study used procedure-related outcomes as indicators of skill acquisition, as it did not differentiate between supervision levels. Our analysis shows that the risk of reoperation on for recurrent hernia was not associated with the number of procedures performed. Higher complication rates were, however, associated both with a low number of procedures performed and the absence of supervision, further emphasizing the benefits of direct supervision, especially during early training.

The learning curve of a surgical procedure can be assessed through outcome measurements or parameters related to skill development^26,27^. In previous studies, procedure-based assessment scores and stabilization of operation time were used to indicate learning progression. One study found that independent competency for groin hernia repair was achieved after 64 procedures^28^, while another study observed stabilization of operation time after 37–42 procedures^29^. According to the Dreyfus and Dreyfus skill acquisition framework, a level-four trainee can intuitively handle diverse scenarios. In our study, surgical complications decreased after approximately 60 procedures, indicating potential attainment of level four in the Dreyfus and Dreyfus framework. As mean operation time and complications continued to decrease, further progress on the learning curve is expected beyond the initial 60 procedures.

The present study suggests that 60 procedures during surgical residency could be a reasonable target when residents are expected to perform this procedure independently after graduation. Nonetheless, it is essential to acknowledge the complexity and individual variability in the learning process of surgical trainees. Individual assessments should decide a trainee’s readiness to perform this procedure independently rather than number of procedures performed.

## Supplementary Material

zrad108_Supplementary_Data

## Data Availability

Deidentified data will be made available upon request by other researchers.
