# Supplementary material for: Learning curve in open groin hernia surgery: nationwide register-based study
Source: BJS Open. 2023 Oct 26;7(5):zrad108. doi: 10.1093/bjsopen/zrad108 (PMC10601449; doi:10.1093/bjsopen/zrad108)

**Learning curve in open groin hernia surgery: nationwide register-based study**

**Authors**

Olof Bladin MD^1^, Nathalie Young MD^2,3^, Jonas Nordquist PhD^4^, Joy Roy MD PhD^1^, Hans Järnbert-Pettersson PhD^3^, Gabriel Sandblom MD PhD^3,5^ and Jenny Löfgren MD PhD^1^

**Author affiliations**

1. Department of Molecular Medicine and Surgery, Karolinska Institute, Stockholm, Sweden.
2. Acute and Trauma Surgery, Karolinska University Hospital, Stockholm.
3. Department of Clinical Science and Education, Södersjukhuset Karolinska Institute, Stockholm, Sweden.
4. Department of Medicine (Huddinge), Karolinska Institute, Stockholm, Sweden.
5. Department of Surgery, Södersjukhuset, Stockholm.

**Correspondence to**

Olof Bladin, MD, Department of Molecular Medicine and Surgery, Karolinska Institute, Karolinska University Hospital, Solna (L1:00), Anna Steckséns gata 53, SE-171 76 Stockholm, Sweden. Telephone: +46 730490933. Fax +46 08-311101 (Karolinska Institute secretary department).

Email: [Olof.bladin@ki.se](mailto:Olof.bladin@ki.se)

**Supplementary Materials - Index**

| **Supplementary Figures and Tables** |  |
| --- | --- |
| Supplementary Figure 1. Tree analysis of operation times. | *page 2* |
|  |  |

**Supplementary Figures and Tables**

Supplementary Figure 1.

Legend: Tree analysis of operation times.


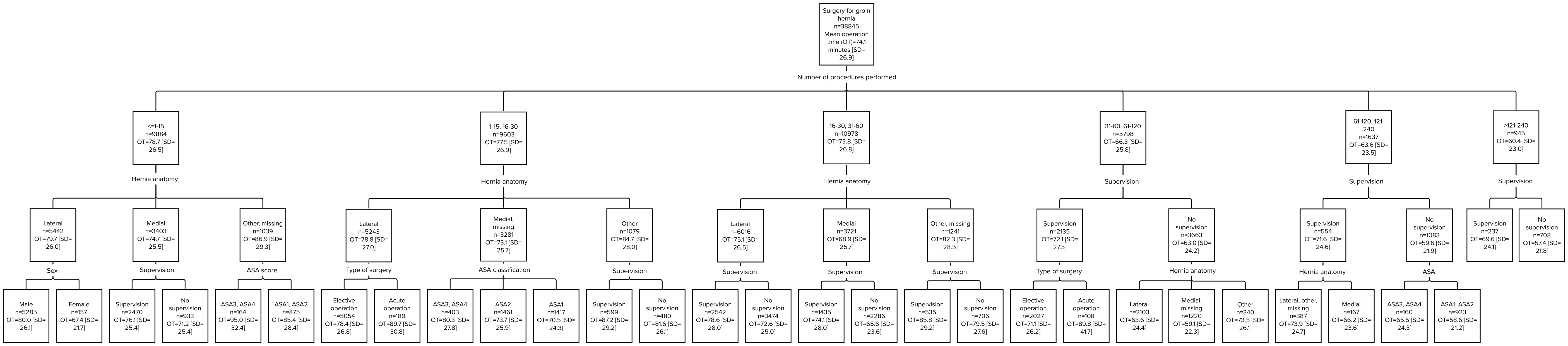

Supplement: zrad108_Supplementary_Data [file zrad108_supplementary_data.docx]
